# The impact of age and oral calcium and vitamin D supplements on postoperative hypocalcemia after total thyroidectomy. A prospective study

**DOI:** 10.1186/1471-2482-13-S2-S11

**Published:** 2013-10-08

**Authors:** Salvatore Tolone, Ruggiero Roberto, Gianmattia del Genio, Luigi Brusciano, Domenico Parmeggiani, Vincenzo Amoroso, Giuseppina Casalino, Ignazio Verde, Alfonso Bosco, Antonio D'Alessandro, Raffaele Pirozzi, Gianluca Rossetti, Paolo Limongelli, Ludovico Docimo

**Affiliations:** 1 Division of General and Bariatric Surgery, Department of Surgery, Second University of Naples, Via S. Pansini 5, 80131, Naples, Italy

## Abstract

**Background:**

Hypocalcemia caused by transient or definitive hypoparathyroidism is the most frequent complication after total thyroidectomy (TT). We aimed to compare the impact of age and the clinical usefulness of oral calcium and vitamin D supplements on postoperative hypocalcemia after TT, and to determine which risk factors are important for hypocalcemia incidence.

**Methods:**

Two hundred consecutive patients treated by TT were included prospectively in the present study. All patients supplemented oral calcium and vitamin D in the post-operative time. The data concerning symptomatic and laboratoristichypocalcemia were collected.

Patients were evaluated according to age, sex, postoperative serum calcium levels, and preoperative serum alkaline phosphatasis levels.

**Results:**

Symptomatic hypocalcemia developed only in 19 patients (9.5%), whereas laboratory hypocalcemia developed in 36 patients (18%). The risk for postoperative hypocalcemia was increate 20-fold for patients older than 50 years.

**Conclusions:**

Age is significantly associated with postoperative hypocalcemia. Implementing oral calcium and vitamin D after total thyroidectomy can reduce the incidence of hypocalcemia related to surgery.

## Background

Total thyroidectomy (TT) is now the preferred option for the management of benign multinodular goiter. Postoperative hypocalcemia is observed in up to one third of total or completion thyroidectomy patients and is the most common complication, due to parathyroid gland insufficiency, and it continues to challenge even experienced surgeons since it often extends the duration of hospital stay and increases the need for biochemical tests [1-5]. Surgical technique has evolved to preserve parathyroid function wherever possible. However, transient hypoparathyroidism still occurs owing to parathyroid manipulation, devascularization, venous engorgement or inadvertent removal of the parathyroid glands with the thyroid specimen.

Hypocalcemia after total thyroidectomy is usually transient, and the incidence of permanent hypoparathyroidism is 3% or less according to the experience of most of the surgical units studied [6-8]. Despite being self-limiting in most patients, symptomatic hypocalcemia is of particular concern because of a delay in its manifestation and the consequent need for prolonged patient hospitalization or readmission. Following total thyroidectomy, patients are closely observed for bleeding in the first 24 h. The main discharge-limiting factor thereafter is the development of hypocalcaemia as patients not at risk of hypocalcaemia may be discharged on day 1 following surgery.

The causes of hypocalcemia after TT are multifactorial, and some of these factors include iatrogenic surgical trauma to the parathyroid glands, incidental parathyroidectomy, the number of functioning glands left behind, extent of surgery, experience of the surgeon, hyperthyroidism, retrosternal goiter, concomitant neck dissection, and thyroid carcinoma [9,10]. Several authors have attempted to identify risk factors in the development of hypocalcemia. Declines in serum calcium [7] or intact parathyroid hormone (iPTH) levels [11,12] after surgery have been suggested as being reliable predictors of postoperative hypocalcemia. Although measurements of serum calcium or iPTH allow the identification of patients who have no risk of hypocalcemia after total thyroidectomy, it is not always easy to predict which patients can be discharged early from the hospital or to identify those requiring close monitoring of serum calcium levels or those that should receive calcium and vitamin D supplements. Implementation of protocols using postoperative PTH measurement has been shown to facilitate day 1 discharge after thyroidectomy [13]. However, rapid PTH measurement is not readily available in many hospitals.

Routine oral calcium and vitamin D supplements have been proposed to prevent the development of symptomatic hypocalcemia and to increase the likelihood of early hospital discharge after bilateral surgical treatment of the thyroid gland or exploration of the parathyroid glands [14,15]. Vitamin D plays a critical role in calcium homeostasis [16]. Insufficiency in calcium absorption due to low vitamin D concentration leads to an increase in parathyroid hormone (PTH) secretion. Increased PTH stimulates the synthesis of calcitriol and thereby improves calcium absorption efficiency. However, even in patients who practiced a postoperative implementation with oral calcium and vitamin D, the risk of postoperative hypocalcemic crisis is not avoided, suggesting a role for other risk factors. Identifying some of this risk factors can led, in turn, to a reduction in costs associated with multiple blood samplings in monitoring the development of hypocalcemia as well as costs associated with prolonged hospitalization.

The aims of this prospective clinical study are: (1) to compare the impact of age and the clinical usefulness of oral calcium and vitamin D supplements on postoperative hypocalcemia after TT, and (2) to determine which risk factors are important for hypocalcemia incidence.

## Methods

In the setting of a prospective study, 200 consecutive patients [44 men (22%, mean age 45 ± 14 years), 156 women (78%, mean age 43±18 years)], undergoing total thyroidectomy in the Division of General and Bariatric Surgery at University of Naples S.U.N. between March 2011 and February 2013, were enrolled. All of the patients had no history of prior thyroid or neck surgery. Patients requiring unilateral lobectomy or subtotal or completion thyroidectomy were excluded, and only patients undergoing total thyroidectomy were enrolled into the study. All patients had normal renal function at the time of surgery. None of the patients had signs or symptoms indicating metabolic bone disease, and none of the patients were on medications, such as oral calcium/ vitamin D supplementation, anti-resorptive agents, hormone replacement therapy for postmenopausal women, anabolic agents, thiazide type diuretics, or antiepileptic agents, known to affect serum calcium metabolism. Indications for surgery are listed in Table 1; each subject provided a specific informed consent, before being part of the study. The study plan was reviewed and approved by local institutional ethical committee.

**Table 1:** Preoperative diagnosis

Disease	TOTAL N = 200
**Multinodular goiter**	168 (84%)
**Carcinoma**	20 (10%)
**Basedow**	12 (6%)

The TT procedure consists of a 3- to 5-cm skin incision 1 to 1.5 cm above the sternal notch. After division of the platysma, the cervical lineaalba is opened without division of the strap muscles. The thyroid lobe is dissected progressively from the strap muscles. After identification of the recurrent laringea nerve and parathyroid glands, the vascular pedicles of the thyroid lobe are ligated with the Harmonic Ace/Focus scalpel (Ethicon Endo-Surgery Inc., Cincinnati, OH, USA), and the thyroid lobe is removed [17-20]. After a check for hemostasis, a drain is placed in the thyroid bed. The cervical lineaalba and platysma are sutured with absorbable sutures, and the skin is closed by an intracutaneous running suture.

Patients were asked to take oral calcium 2 g/d taken twice (1 g every 12 hours) and vitamin D 1 g/d taken twice (0.5 g every 12 hours) from the night of operation to post-operative day 14. Intravenous calcium gluconate was administered if significant symptomatic hypocalcemia persisted after surgery despite oral supplementation.

The medical and nursing notes were carefully examined for documentation of symptoms of hypocalcaemia. The time of administration of calcium, vitamin D analogues and intravenous fluids in relation to serum calcium and hypocalcaemic symptoms was also noted. Serum calcium, albumin, creatinine, and alkaline phosphatase levels were determined the day before surgery, on the evening of surgery ('day 0'), on the morning of day 1 and then every 24 hours until patient discharge. Serum calcium concentration was corrected for changes in serum albumin. Serum calcium, creatinine, albumin, and alkaline phosphatase were measured using automated assays. The reference ranges for serum calcium in our laboratory were 8.5 to 10.5 mg/dL.

All patients were seen in the outpatients department 2 weeks after surgery. The reported symptoms of hypocalcaemia and the treatment required to control these symptoms were noted.

Postoperative hypocalcemia was defined as either symptomatic or laboratory.

Hypocalcemic symptoms and signs, from perioral tingling and numbness to carpopedal spasms and tetany, were registered in detail. Laboratory hypocalcemia was defined as serum total calcium concentrations of <8.0 mg/dL, even if recorded only in a single measurement.

### Statistics

Data were analyzed using SPSS 15.0 for Windows (SPSS Inc, Chicago, IL). Results were expressed as mean ± SD. Comparisons of data used the Wilcoxon signed-rank test, chi-square test, and logistic regression analysis. Results were considered statistically significant when the 2-tailed *P *value was less than 0.05.

## Results

All patients respected the oral supplement protocol. The thyroid diseases of the patients included in the study are shown in Table 1. The operative procedures were all total thyroidectomy. No major complication occurred (e.g. persistent vocal cord paralysis and hypoparathyroidism, bleeding requiring reintervention). Incidental parathyroidectomy was found in 3 patients (1.5%).

The means (±SD) for serum albumin, creatinine, calcium and alkaline phosphatase levels were 3.8 ± 0.09 g/dL, 0.8 ± 0.1 mg/dL, 8.9 ± 0.2 mg/dl, and 162 ± 74 U/L, respectively. The postoperative serum calcium level was lower than the preoperative serum calcium level (8.9 ± 0.2 mg/dLvs 8.2 ± 0.9 mg/dL, *P *< 0.001).

Symptomatic hypocalcemia developed only in 19 patients (9.5%), whereas laboratory hypocalcemia developed in 36 patients (18%). Hypocalcemic symptoms were minimal in 12 patients. Intravenous calcium was administered to 7 patients with severe hypocalcemic symptoms (Table 2). Regarding these latter cases only, in the subsequent hystopathological analysis on removed thyroid tissue, incidental parathyroid tissues were found in three. Permanent hypocalcemia developed in none of patients. Hypercalcemia or other side effects did not develop in any of the patients receiving routine oral supplements.

**Table 2:** Patients developing hypocalcemia

Hypocalcemia	Total N = 200
LaboratoryHypocalcemia	36 (18%)
SymptomaticHypocalcemia	19 (9.5%)
Requiring E.V. Calcium	7 (3.5%)

Age and serum alkaline phosphatase levels were significantly higher in patients who developed hypocalcemia than in others (56.7 ± 8 years and 242.21 ± 72 U/L vs 42.1 ± 9 years and 123.21 ± 41 U/L, *P *< 0.001). Postoperative serum calcium levels were significantly lower in hypocalcemic patients (7.3 ± 0.4 mg/dLvs 8.6 ± 0.3 mg/dL, *P *< 0.001).

Hospital stay was significantly longer in hypocalcemic patients (4 ± 1 days, range 2-5 days) than others (2 ± 0.5 days, range 1-3 days) (*P *< 0.001). In female patients, the ratio of postoperative hypocalcemia was found to be significantly higher when compared to male patients (24% vs 8%, *P <*0.01).

There was a negative correlation between serum calcium level and age (*r*s- 0.501, *P *< 0.001) and female gender (*r*s- 0.203, *P *< 0.01) and serum alkaline phosphatase level (*r*s-0.498, *P *< 0.001).

Patient age and preoperative serum alkaline phosphatase level, were independent significant variables in the development of hypocalcemia after thyroidectomy. The risk for postoperative hypocalcemia was increased 20-fold for patients older than 50 years (odds ratio [OR] 20.2; 95% confidence interval [CI] 10.4- 58.3).

## Discussion

The present study shows that post-operative oral calcium and vitamin D supplements can reduce the incidence of hypocalcemia after total thyroidectomy. Also, we investigated the prediction value of age, preoperative serum calcium and alkaline phosphatase on postoperative hypocalcemia after total thyroidectomy.

We found that age was significantly associated with postoperative hypocalcemia. Negative correlations were observed between the serum calcium level and age, and serum alkaline phosphatase level. According to a logistic regression analysis, age was a significant independent variable associated with postoperative hypocalcemia.

Postoperative hypocalcemia is one of the most frequent complications after TT [21]. In several studies, the incidence of hypoparathyroidism varied from 1.6% to above 50% [7,22].

The causes of hypocalcemia after TT are multifactorial, and some of these causes include injury, devascularization, and unintentional excision of parathyroid glands, the number of functioning glands left behind, the extent of surgery, the experience of the surgeon, hyperthyroidism, retrosternal goiter, concomitant neck dissection, and thyroid carcinoma [22-27].

However, the role of implementation of oral calcium and vitamin D is not new, and it is considered an effective approach to the prevention of hypocalcemia.

Infact, this treatment prevented a significant decrease of serum calcium levels as well as the subsequent development of major hypocalcemic symptoms after total thyroidectomy. The symptoms reported by patients were minimal and only seven required intravenous calcium administration after his persistent significant hypocalcemia failed to respond to implementation of oral calcium and vitamin D. However, in this case hypocalcemic status was probably due to the accidental removal of parathyroid tissue during surgery, as shown by hystological analysis.

Two studies have evaluated the efficacy of routine calcium supplements for the prevention of hypocalcemia after thyroidectomy [28,29]. Moore [28] reported that only 4 of 124 patients who received daily treatment of calcium (5 g) after bilateral thyroid resection developed hypocalcemia, and 1 required administration of intravenous calcium. Based on empirical observations, the prophylactic use of oral calcium to reduce the risk of hypocalcemic crisis and increase the likelihood of early hospital discharge was recommended. Bellantone et al [29] reported in a prospective control study that only 3 of 26 patients (11%) receiving oral calcium supplement (3 g/d) had symptoms related to hypocalcemia after total thyroidectomy, whereas 11 of 27 patients (40%) not receiving calcium supplement had symptoms. These studies suggest that postthyroidectomyhypocalcemia can be considerably prevented by the routine administration of calcium supplements.

The risk for postoperative hypocalcemia for patients undergoing TT was higher than that for patients with advanced age.

Recently, there has been a great deal of interest in identifying perioperative factors that can predict the development of hypocalcemia after thyroidectomy [30,31]. The drive towards a shorter hospital stay following total thyroidectomy has led to a number of studies evaluating the use of plasma biochemical markers to predict the development of hypocalcaemia. Thyroid surgery impairs PTH secretion by the parathyroid glands resulting in postoperative parathyroid insufficiency and subsequent development of hypocalcaemia [32]. The measurement of serum calcium concentration following thyroidectomy has been used in an attempt to predict the development of hypocalcaemia. However, the measurement of total serum calcium is inaccurate, at least in part, owing to postoperative haemodilution [33,34].

The value of PTH in predicting post-thyroidectomy hypocalcemia has been extensively investigated and reported in the literature [35,36] Although the use of PTH post-thyroidectomy may allow shorter hospital admission, rapid access to results of postop- erative PTH measurement is not widely available in most hospitals. In addition, there is no consensus on the threshold for PTH and the optimal timing for its measurement post-thyroidectomy. The PTH measurement on the first postoperative day has been shown to be a useful method to predict post-thyroidectomy hypocalcemia [37]. Serum PTH concentration, when checked 1-6 h after thyroidectomy, has a higher accuracy in predicting hypocalcaemia [38]. Whilst the use of serum PTH levels to predict post-thyroidectomy hypocalcaemia is well established, it appears to lack the desirable 100% accuracy rate. We were not able to analyse the postoperative percentage decline in PTH owing to the lack of preoperative PTH data. However, both absolute levels and percentage decline have been used to predict hypocalcaemia with similar accuracy, and, although similar, levels vary between institutions [36].

Also, we think that post-operative hypocalcemia can be reduced when a precise minimal surgical dissection is performed; in fact, the use of harmonic scalpel can help to avoid energy spread, blood loss and edema, thus limiting parathyroid function impairment [17-20].

Vitamin D has a critical role in calcium homeostasis. Vitamin D deficiency has been frequently documented in medically ill and free-living populations [39,40] Advanced age is reported to be a major risk factor for vitamin D deficiency. Aging is associated with alterations in vitamin D metabolism. Studies have observed (1) an age-related decrease in renal 1-hydroxylase activity and intestinal calcium absorption, and (2) an age-related decline in the cutaneous accumulation of 7-dehydrocholesterol, which is converted into previtamin-D3 by solar ultraviolet radiation. Insufficient calcium absorption due to low vitamin D concentrations leads to an increase in PTH secretion. Increased PTH stimulates the synthesis of calcitriol and thereby improves calcium absorption efficiency [39,40].

In previous studies, regimens of oral calcium alone and of a calcium and vitamin D combination were effective. The study from Bellantone et Al [29] also revealed that the addition of vitamin D to oral calcium supplements was associated with significantly higher serum calcium concentrations on post- operative days 2 and 3 and with a lower incidence of hypocalcemia. Although it has been reported that vitamin D administration inhibits iPTH secretion by normally functioning parathyroid glands, prior studies and our own results showed that iPTH secretion was not affected by vitamin D administration in postthyroidectomy patients. Therefore, the early use of vitamin D in addition to calcium supplements can be recommended for patients undergoing total thyroidectomy. The dosages and durations of calcium and vitamin D administration are also of concern. In a study from Roh and Park [32], routine oral calcium and vitamin D supplements were effective in reducing the incidence (6%) and severity of hypocalcemia after total thyroidectomy. Only a minority of patients receiving the supplements presented with minimal symptoms related to hypocalcemia, and higher levels of serum calcium during the first few days after total thyroidectomy were measured in patients receiving the supplement.

In a previous study, we also documented that regimens of oral calcium alone and of a calcium and vitamin D combination were effective in most cases of thyroidectomy performed for benign thyrodeal pathology [41-44].

## Conclusions

The prevention of significant symptomatic hypocalcemia will allow early discharge of postthyroidectomy patients from the hospital. In turn, early discharge eliminates the necessity of multiple blood samplings for close monitoring of serum calcium or iPTH levels. However, the risk of postoperative hypocalcemia for patients undergoing TT was higher than that for patients with advanced age. In these patients, an accurate clinical and laboratoristic follow up during the post-operative period is necessary.

In conclusion, our data suggest that postoperative oral calcium and vitamin D supplements can take a role the prevention of postoperative hypocalcemia and for increasing the likelihood of a safe and early discharge from the hospital.

## List of abbreviations

TT: Total Thyroidectomy; PTH: Parathyroid Hormone; iPTH: intact Parathyroid Hormone

## Competing interests

The authors declare that they have no competing interests.

## Authors' contributions

ST, RR, PL have made substantial contributions to conception and design; RP, GR have made substantial contributions to acquisition of data; GC, AB, VA, ADA have made substantial contributions to analysis and interpretation of data; ST, RR, IV have been involved in drafting the manuscript; RR, PL, GDG, LB, DP have been involved in revising it critically for important intellectual content; ST, RR and LD have given final approval of the version to be published.

## Authors' information

ST: Research Grant Fellow at Second University of Naples; RR: Assistant Professor of Surgeryat Second University of Naples; GDG: Assistant Professor of Surgeryat Second University of Naples; LB: Senior Doctorat Second University of Naples; DP: Assistant Professor of Surgeryat Second University of Naples; VA: Surgical FellowResidentat Second University of Naples; GC: Surgical FellowResidentat Second University of Naples; IV: Senior Doctorat Second University of Naples; AB: Surgical Fellow Residentat Second University of Naples; ADA: Surgical Fellow Residentat Second University of Naples; RP: Surgical FellowResidentat Second University of Naples; GR: Senior Doctorat Second University of Naples; PL: Assistant Professor of Surgeryat Second University of Naples; LD: Full Professor of Surgeryat Second University of Naples.

## Declarations

Funding of this article has come from personal funds.

This article has been published as part of *BMC Surgery *Volume 13 Supplement 2, 2013: Proceedings from the 26th National Congress of the Italian Society of Geriatric Surgery. The full contents of the supplement are available online at http://www.biomedcentral.com/bmcsurg/supplements/13/S2
